# Wild harbour porpoises startle and flee at low received levels from acoustic harassment device

**DOI:** 10.1038/s41598-023-43453-8

**Published:** 2023-10-04

**Authors:** Siri L. Elmegaard, Jonas Teilmann, Laia Rojano-Doñate, Dennis Brennecke, Lonnie Mikkelsen, Jeppe D. Balle, Ulrich Gosewinkel, Line A. Kyhn, Pernille Tønnesen, Magnus Wahlberg, Andreas Ruser, Ursula Siebert, Peter Teglberg Madsen

**Affiliations:** 1 Zoophysiology, Dept. of Biology, Aarhus University, 8000 Aarhus, Denmark; 2 Marine Mammal Research, Dept. of Ecoscience, Aarhus University, 4000 Roskilde, Denmark; 3 Institute for Terrestrial and Aquatic Wildlife Research, University of Veterinary Medicine Hannover, Foundation, 25761 Büsum, Germany; 4 Norwegian Polar Institute, 9296 Tromsø, Norway; 5 Environmental Microbiology, Dept. of Environmental Science, Aarhus University, 4000 Roskilde, Denmark; 6 Marine Biological Research Centre, Dept. of Biology, University of Southern Denmark, 5300 Kerteminde, Denmark

**Keywords:** Behavioural ecology, Ecophysiology

## Abstract

Acoustic Harassment Devices (AHD) are widely used to deter marine mammals from aquaculture depredation, and from pile driving operations that may otherwise cause hearing damage. However, little is known about the behavioural and physiological effects of these devices. Here, we investigate the physiological and behavioural responses of harbour porpoises (*Phocoena phocoena*) to a commercial AHD in Danish waters. Six porpoises were tagged with suction-cup-attached DTAGs recording sound, 3D-movement, and GPS (n = 3) or electrocardiogram (n = 2). They were then exposed to AHDs for 15 min, with initial received levels (RL) ranging from 98 to 132 dB re 1 µPa (rms-fast, 125 ms) and initial exposure ranges of 0.9–7 km. All animals reacted by displaying a mixture of acoustic startle responses, fleeing, altered echolocation behaviour, and by demonstrating unusual tachycardia while diving. Moreover, during the 15-min exposures, half of the animals received cumulative sound doses close to published thresholds for temporary auditory threshold shifts. We conclude that AHD exposure at many km can evoke both startle, flight and cardiac responses which may impact blood-gas management, breath-hold capability, energy balance, stress level and risk of by-catch. We posit that current AHDs are too powerful for mitigation use to prevent hearing damage of porpoises from offshore construction.

## Introduction

Increasing anthropogenic noise in the oceans is of growing concern due to the adverse effects noise may inflict on marine life^1^. Most anthropogenic noise is produced incidentally by activities at sea, but in some cases noise is produced purposefully to deter marine mammals from specific locations or areas. The most common example is the noise produced by Acoustic Harassment Devices (AHD, source level > 185 dB re 1 µPa @ 1 m root mean squared, rms^2^) and Acoustic Deterrent Devices (SL < 185 dB re 1 µPa rms^2,3^), also known as ‘seal scarers’, which have been developed to deter pinnipeds from fisheries and aquaculture to prevent depredation and damage to fishing gear^4^. While the efficacy of the largely unregulated AHDs in terms of scaring seals away from lucrative sources of concentrated food is debated^3^, mounting evidence suggest that AHD sounds may have substantial negative collateral effects on sympatric species such as harbour porpoises^5–8^.

Data from passive acoustic monitoring (PAM) and visual tracking show that detections of porpoises decrease out to a range of 7.5 km when AHDs are active^4,6^. Such observations of porpoise avoidance have prompted an increased use of AHDs prior to very loud marine activities, such as pile driving, to deter harbour porpoises to a safe distance, thereby mitigating risk of temporary or permanent hearing threshold shifts (TTS or PTS)^9^. It remains unknown however, how individual harbour porpoises respond to specific received levels (RL) of these pulses, and at which RL and hence range the desired deterrence effect is achieved. This information is critical in relation to activities such as offshore construction, since the deterrence sound must be efficient in clearing an area of porpoises, yet it should not cause disruptions at a scale comparable to the pile driving itself, let alone inflict hearing impairment^8^. This concern is warranted since many AHDs employ source levels (SL) up to 193 dB re 1 µPa (rms) @ 1 m at main frequencies of 10 to 15 kHz^3,8^, where porpoises hear well^10^. A meta-analysis of exposure studies suggests that porpoises respond behaviourally at RL 45 dB above their hearing threshold at a given frequency^11^. Accordingly, the predicted response threshold for a 14 kHz AHD is ~ 95 dB re 1 µPa rms, given their corresponding hearing threshold of ~ 50 dB re 1 µPa rms^10^. The high SLs of AHDs implies that almost 100 dB of transmission loss is needed before the signal has decreased to the response threshold of ~ 95 dB, resulting in potentially very large effect ranges.

While studies show a decreased presence of porpoises in relation to active AHDs^5–7,9,12^, detailed information on the behavioural and physiological responses of individual cetaceans to AHDs is non-existent to our knowledge. Porpoises and other prey species may have evolved innate anti-predator responses to sounds akin to those of their predator species. Given the similarity to killer whale vocalisations (frequency range from below 1 kHz to 20 kHz^13,14^), tonal sounds from sonars or AHDs may thus trigger innate defence responses in prey species such as harbour porpoises. AHDs may therefore provoke costly anti-predator responses^15,16^, such as freezing in silence^17^ or flight^18^. Furthermore, the high SL and fast rise times of AHD pulses may evoke acoustic startle responses^19^ as recently documented in captive odontocetes including harbour porpoises^20–22^. In the startle response, muscles of the whole body flinch instantly as a pre-cognitive protective measure from potentially harmful sudden stimuli^3,23^, and may be accompanied by heart rate fluctuations^24^.

Energetic responses to noise may have a cumulative impact on health if they occur frequently enough and especially in populations that already have impaired health status^25,26^. But direct harm is also possible from some physiological responses: studies have shown that porpoises, dolphins and belugas in human care may alter cardiovascular regulation in response to sound exposure, either with increased or decreased heart rate, depending on sound type, individual, context, perceived threat and naivety^22,27–29^. Changes in heart rate and tissue perfusion at water depths with high pressure, but not sufficiently high to cause lung collapse, may increase the risk of decompression sickness, since diminished or augmented gas fluxes between tissues, blood and lungs can either trap nitrogen in supersaturated tissues, or potentially contribute to supersaturation. Both scenarios would increase the risk of gas bubble formation^30,31^, and may explain mass-strandings of beaked whales with gas-bubble lesions following naval sonar exercises^32,33^ as well as North Sea porpoises with gas emboli^34^. Additionally, an increased heart rate and tissue perfusion would enhance oxygen delivery and blood oxygen depletion, putting an individual at risk of asphyxia. Direct fitness consequences may also arise as collaterals from behavioural changes that increase risk of strandings or bycatch.

This study aims to help inform the future use of AHDs by exploring detailed behavioural and physiological responses of wild harbour porpoises to AHD exposures as a function of actual RL recorded on the animal with acoustic tags. Specifically, we test the hypothesis that low to moderate AHD exposure induces a rapid change in behaviour and heart and respiratory rate that may have implications for harbour porpoise health and population fitness.

## Methods

### Tagging and AHD exposure

Six harbour porpoises were tagged during 2018 and 2019 after incidentally being trapped in pound nets set by local fishermen in Danish waters^35^ (Table 1). Tagging was carried out within 24 h after discovering the porpoise, during which time the porpoises could swim and breathe freely inside the boundaries of the small-mesh net (2 × 2 cm). The animals were carefully lifted on-board a small boat and placed on a soft pad where sex was determined, morphometric measurements taken and blubber thickness assessed with ultrasound probes. Blubber thickness measures were within the reported range of 1.5–2.5 cm for porpoises in the same areas^36^. Porpoises were handled on the boat for a maximum of 19 min (mean 9 min). At variable times after release, porpoises were exposed to 15 min of a commercial AHD (Lofitech, Leknes, Norway) playing 500 ms 14 kHz pulses (hereafter; pings) at random intervals of 0.6–90 s^7^. Each ping has a source level (SL) of 189 dB re 1 µPa (rms) @ 1 m or sound exposure level (SEL, 500 ms) of 184 dB re 1 µPa^2^s measured 360° around the transducer in the horizontal plane. The AHD transducer hung from the boat at 2-m depth during playback. In 2018, the exposures were conducted from the tagging boat and location, starting 16–17 min after release. We aimed to expose the animals at about 1 km (the usual distance goal of mitigation deterrence), and estimated that if the porpoises had a travel speed of 1.5 m s^−1^ in a straight line away from the tagging site^37^, this distance would be achieved after ~ 11 min. We waited 15 min considering that the porpoise might pause or change direction. In 2019, exposures started 90–202 min after release to achieve lower AHD received levels at longer ranges. After tagging, the porpoises were tracked with 3-element VHF antennae (~ 220 MHz VHF-transmitters were built into the Dtags) from a separate tracking boat to assess travel direction and distance and to eventually expose animals from a location and angle different from the tagging site.

**Table 1:** Information on the tagged animals exposed to AHD.

Animal ID	Deployment ID	Type of DTAG	Deployment date	Age class and sex	Standard length (cm)	Blubber thickness (mm)	Hours of data (hours of ECG data)	Initial AHD RL (dB re 1 μPa rms)	Initial distance to AHD (km)	Time from release to exposure (minutes)	Time from release until feeding buzzes occur (minutes)	Time from exposure end until feeding buzzes occur (minutes)
HP1	hp18_134a	DTAG4 (GPS)	14-05-2018	Juvenile F	111	22	44	130	0.9	16	–	34
HP2	hp18_151a	ECG-DTAG3	31-05-2018	Juvenile M	122	16	10 (2.5)	131	–	17	–	24
HP3	hp19_262a	DTAG4 (GPS)	19-09-2019	Juvenile M	115	17	37	132	2.2	202	19	16
HP4	hp19_289a	ECG-DTAG3	16-10-2019	Juvenile M	117	18	14 ( 3)	98	–	189	24	42
HP5	hp19_290a	DTAG4 (GPS)	17-10-2019	Juvenile M	112	16	2	99^a^	6.9	100	75	Tag off^b^
HP6	hp19_290b	DTAG3	17-10-2019	Juvenile F	132	19	12	106	–	90	22	25

Age class determined according to Lockyer and Kinze^73^. Blubber thickness was measured by ultrasound in 3 lateral and 3 dorsal places on the porpoise. The number given is the average of those 6 measurements for each animal.

^a^HP5 generally had low signal-to-noise ratios, so the initial five pings used for RL evaluation were found among the initial 34 pings.

^b^Tag fell off 4 min after end of exposure, and the porpoise had not yet started feeding during this time.

The handling, tagging and acoustic exposure of harbour porpoises was performed in accordance with the Danish law. The specific permissions were evaluated and issued by the Environmental Protection Agency under Ministry of Environment of Denmark (permission number SVANA-610-00118, https://eng.mst.dk/) and by The Animal Experiments Inspectorate under Ministry of Food, Agriculture and Fisheries of Denmark (permission number 2015‐15‐0201‐00549, https://en.dyreforsoegstilsynet.dk/). The handlers of the porpoises had FELASA certification (Federaton of Europoean Laboratory Animal Science Association, category B).

### Tags

The animals were equipped with either a DTAG3 (on HP6), an ECG-DTAG3 (on HP2 and HP4) that also measured heart rate, or a DTAG4 (on HP1, HP3, and HP5) with a GPS receiver (Table 1). All tags were attached dorsally on the porpoises with four suction cups approximately 5 cm behind the blowhole to ensure good quality acoustic recordings of the echolocation and respirations of the tagged animal, without the risk of mistaking signals from conspecifics. The tags measured no more than 7 × 17 × 3.5 cm and weighed 221–321 g. The tags sampled 16-bit stereo audio at 500 kHz (ECG-DTAG3), 240 kHz (Mid-frequency DTAG3), or mono audio at 576 kHz (DTAG4) (175 dB re 1 µPa clip-level; with a flat (± 3 dB) frequency response at 0.5–150 kHz). The tags also recorded three-dimensional acceleration (200–625 Hz, 16-bit) and magnetometer data (50–625 Hz, 16-bit), as well as pressure (50–625 Hz, 16 bit). The ECG-DTAG3 additionally measured electrocardiogram (5 kHz sampling, 16-bit resolution and a 2-pole, 200 Hz anti-alias filter) with two external differential electrodes (and a salt-water ground) attached to the animal with suction cups along the ventricular contraction axis^38,39^. The tags released incidentally from the animals after 2–44 h.

### Tag data processing and analysis

Tag data were processed using open-source tools (http://www.animaltags.org) and custom-written scripts in MatLab (The MathWorks, Natick, MA, USA). Tag placement on the animals influences the recordings, so it was checked for all animals that the tags did not slide during the compared time intervals. This was checked by examining the three data channels of acceleration as well as magnetometer data. For one animal the tag slid during the exposure (Fig. S3).

We used the detection of feeding buzzes as a proxy for the return to baseline behaviour after tagging. Feeding buzzes were identified via spectrogram inspection (Hamming window, Fast Fourier Transform size 512, 75% overlap) of the sound recording in consecutive 5 s intervals. Buzzes are defined as rapid sequences of echolocation clicks with inter-click-intervals less than 15 ms and an accompanying rapid change in the acceleration signal (to distinguish them from social calls)^40,41^. Echolocation clicks outside of buzzes (i.e., search clicks) from the tagged animal were detected using a supervised click detector. For HP6, which had a mid-frequency DTAG3, the high frequency porpoise clicks were on the limit of what could be reliably detected, and were not included in analyses. Still, buzzes, which contained perhaps hundreds of clicks, could still be reliably detected in spectrograms for HP6 and were included in the analyses.

For each 15-min exposure, a 15-min pre-exposure interval, immediately preceding the exposure, was used as a control. Click counts during the two 15-min intervals were compared for acute exposure effects. Click apparent output levels were estimated by the peak-to-peak (pp) sound pressure in 20 ms segments surrounding each click detection and reported in dB units (dB re 1 µPa pp)^42^. Sound segments were filtered with an 80 kHz 6-pole high-pass filter prior to level measurement. To detect acute exposure effects on click output level, 95 percentiles of all click levels during exposure and 15-min prior to exposure were used. The 95th percentile was used with the rationale that the maximum click output level is the important parameter for acoustic crypsis. Note that apparent click levels are only relative proxies for source levels, and they are not fully comparable across individuals, since levels depend on the exact tag placement on the animal.

Timing of respirations and AHD pings were marked manually by visual inspection of spectrograms. AHD received levels were measured by first band-pass filtering (10–46 kHz 6-pole band-pass filter) sound segments around each ping to filter out most low-frequency flow noise, as well as high-frequency echolocation, while ensuring the inclusion of energy contained in the first two AHD harmonics at 28 and 42 kHz. Ping recordings were manually checked for noise transients and sufficient signal-to-noise ratios (> 10 dB) for RL evaluations. The rms-fast RL (dB re 1 µPa) was then calculated by taking the square root of mean of the pressure squared over a 125 ms window^43^. The cumulated sound exposure level (SELcum, dB re 1 µPa^2^ s) was calculated from the band-pass-filtered AHD pings as the sum of the squared pressure over 500 ms for all received pulses. When using SELcum to assess hearing impairment, we ignored the unknown recovery of the cetacean auditory system during the 15-min exposure period^44^, thereby potentially overestimating the risk of TTS. The timing of the initial feeding buzzes after tagging and exposure were also marked manually to have a measure for handling/exposure stress, with the assumption that feeding represents an, at least partial, normalisation of behaviour^45^. To avoid sensitivity to single buzzes which may be mis-classified social calls, the fifth feeding buzz after tagging/exposure was taken as marking the resumption of feeding.

An activity measure, Minimum Specific Acceleration (MSA, m/s^2^), was calculated from the 3-axis acceleration data decimated to 25 Hz^46^. The 95 percentiles of MSA over 5-s bins were used as a proxy for swimming effort. To detect rapid movements of the tag associated with foraging events and potential acoustic startle responses, jerk (derived acceleration, m/s^3^) was computed as the norm of the differential of the triaxial acceleration at a downsampled sampling rate of 25 Hz, following Ydesen et al.^47^. The startle muscle flinch was measured by significant peaks in jerk appearing within 0.2 s of receiving the AHD ping (sensu Elmegaard et al.^22^).

GPS positions obtained at irregular intervals (typically every 3–5 min) from the three DTAG4 deployments were used to determine distance between the animal and the AHD, as well as travel direction and horizontal travel speed of tagged animals. Horizontal travel speed was calculated from one GPS position to the previous, excluding a small number of GPS-positions deemed to be erroneous due to unrealistic travel speeds above 5 m s^−1^. This threshold was adopted as being well above the top speeds of visually-tracked porpoises exposed to AHDs (3.2 m s^−1^)^9^. No GPS positions needed to be excluded during the AHD exposure periods, avoiding the risk of not detecting high swimming speeds.

Time spent in visual crypsis was defined as time spent within 2 m of the surface or apparent seafloor. The nearness to seafloor was used for animals with GPS information (HP1, HP3 and HP5) or that made U-shaped dives, which were likely to represent a depth close to the seafloor (HP4, HP6). We verified the assumption that U-shaped dives are close to the seafloor using the animals with GPS-positions.

Two deployments included ECG-data that were down-sampled to 250 Hz and filtered (4 pole band pass between 1 and 10 Hz) for automatic detection of R-peaks in the QRS-complex of each heartbeat, which were then visually verified sensu Elmegaard et al.^48^. Instantaneous heart rate (*f*_H_) was calculated from the time difference between an R-peak and the previous^39^.

### AHD exposure without tagging

A separate exposure protocol was employed to serve as a form of control for the tagging efforts. To test if responses to the AHD exposure were amplified (sensitization) or reduced (habituation) by the recent tagging event, an additional exposure experiment without tagging was carried out in autumn 2019 in shallow waters near Kerteminde, Denmark. Over a period of one month, we went to sea on the five days with flat calm water, using a small vessel and a drone. We used a DJI Phantom 4 Professional v2.0 (P4Pv2, http://www.dji.com, with a Polarpro ND8-PL filter, and a built-in gimbal-stabilised DJI camera) to film behavioural effects of AHD playback on un-tagged, presumably undisturbed, porpoises. The porpoises were approached by a small boat while keeping at least 200 m distance, unless the porpoises came closer themselves. During observations, the boat was anchored with the engine off, while a drone monitored baseline behaviour and then behaviour during AHD or sham/control exposure of a focal porpoise. Only trials with drone recordings of exposure of > 30 s, and/or total drone recording > 60 s were analysed. Due to the limited good weather days, only one exposure trial and five control trials were available for the analysis, while three exposure trials and three control trials were discarded due to losing sight of the animals too quickly. During the exposure trial, the focal porpoise swam close to six other porpoises (see suppl. Video SV1). To accommodate the limited range (< 500 m) of the drone, we used a controlled playback of AHD sound as the high output levels of a commercial AHD would potentially result in hearing impairment over these ranges. AHD playback sound files were made from a field recording of the Lofitech AHD at 1 km distance. A 500 ms ping from this recording was repeated at randomised intervals < 10 s. The SL was 158 dB re 1 µPa rms @ 1 m to achieve RL of ~ 100–120 dB re 1 µPa when the playback boat was at 100–500 m from the focal porpoises (RL = SL – 20 × log_10_(range)). Similar sound files with ping sound pressure reduced by 80 dB (i.e., only detectable within a few metres from the source) were used for control playbacks to control for anything in the playback system itself causing the porpoises to respond. The sound-files were played from a handheld LS-14 field recorder (Olympus, Tokyo, Japan) connected to a custom power amplifier and a spherical transducer (Sonar products HS26, Driffield, UK) suspended at 2-m depth. A calibrated SoundTrap (ST300HF v1.7, Ocean Instruments, Auckland, New Zealand) hanging with the transducer, but at 1-m depth, recorded the outgoing pings.

After the animals had departed the area, we estimated the exposure RL. To do this, we anchored a calibrated SoundTrap at 2 m depth at the playback location and subsequently played the AHD pings from the estimated porpoise position at the start of the exposure (determined from the drone GPS). The SoundTrap recorded sound at 96 kHz sampling rate (16-bit resolution, anti-aliasing filter at 42 kHz) with a clip level of 172 dB re 1 μPa (flat frequency response from 20 Hz to 42 kHz ± 3 dB).

The drone was operated under permit number 5411169 from the Danish Transport, Construction and Housing Authority, and logged UTC time, GPS location, altitude, pitch, and yaw of the camera every 100 ms. A custom-made program, Porpoise Tracker (https://github.com/henrikmidtiby/PorpoiseTracker/) was used to calculate porpoise swimming tracks and speeds, while respirations were visually detected from the video recordings. The locations had a precision of 2 m, and the speed within 1 m/s^49^.

## Results

**Figure 1:**
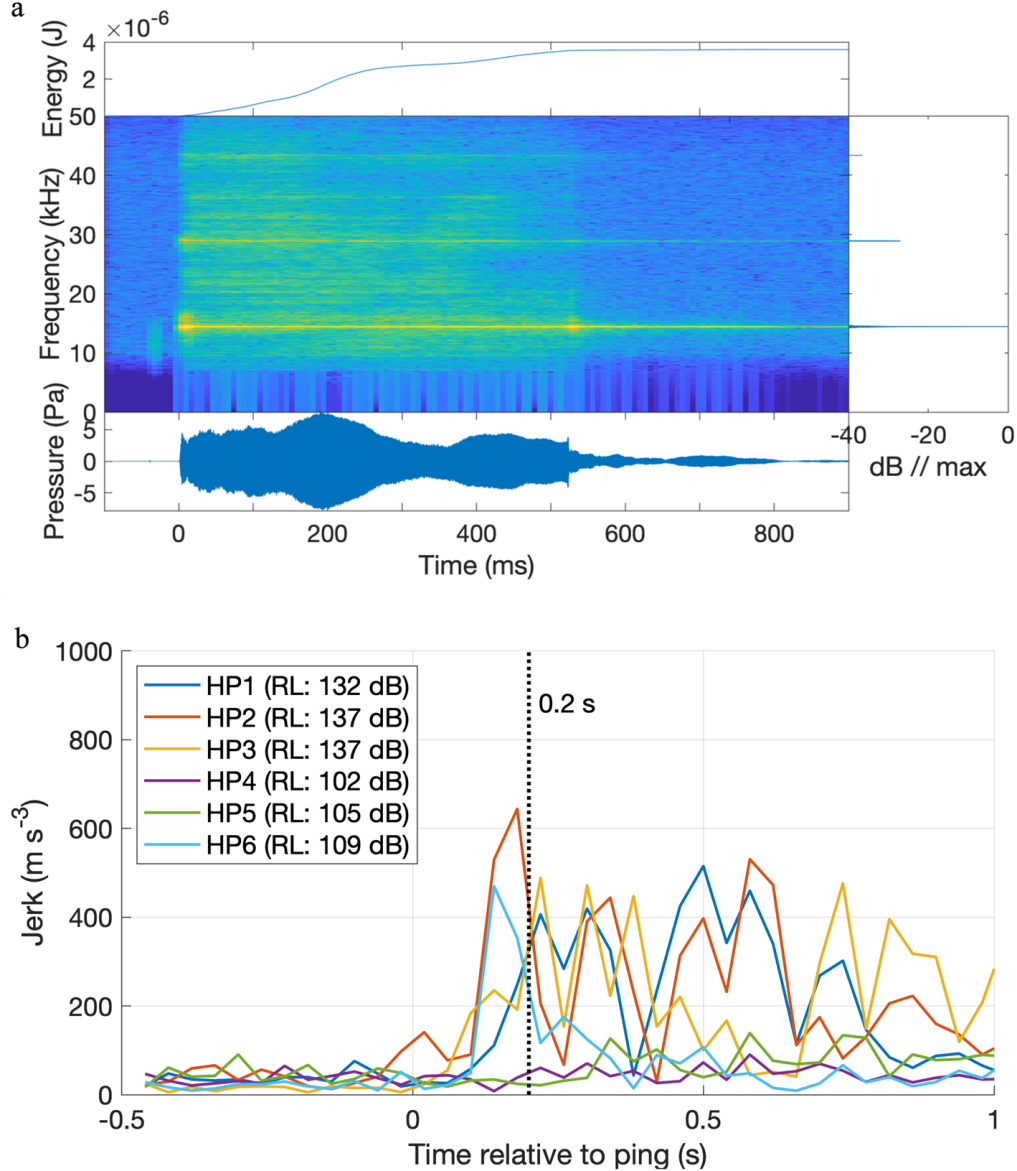
(**a**) AHD ping characterization and (**b**) acoustic startle response in the six tagged porpoises. (**a**) An example of an AHD ping recorded by a DTAG on a porpoise at 1 km distance from the sound source. The bandpass-filtered (10–46 kHz) AHD ping had a received sound pressure level of 132 dB re 1 µPa (rms). The plot depicts the cumulative energy of the ping, the spectrogram, spectral density plot (or power spectrum), and the waveform of the ping. Due to propagation properties of the shallow water at this exposure site, the received ping had a duration of more than a second, although the emitted duration was 500 ms. (**b**) The acoustic startle response was evoked by the AHD pings in four porpoises (HP1,2,3,6): sudden muscle flinches, measured by the derived acceleration (jerk), occur within 0.2 s of receiving the first ping. HP4 and HP5 received the lowest sound pressure levels (dB re 1 µPa, rms fast), as indicated by the legend, and did not startle to these pings.

Six harbour porpoises were tagged with suction-cup-attached multi-sensor DTAGs and exposed to a commercial AHD at initial ranges up to 7 km, resulting in measured RLs of 98–132 dB re 1 µPa (rms-fast) for the mean of the first five pings with sufficient signal-to-noise-ratio (see example of ping in Fig. 1a, Table 1). For HP1-HP3 and HP6 the initial RL were calculated from the first five received pings which all had sufficient signal-to-noise ratio. For HP4 the five pings for calculation of initial RL were between the six first pings, while for HP5, the five pings used for initial RL were between the 34 first received pings, due to poorer signal-to-noise ratios. Feeding buzzes were noted as a proxy for returning to normal behaviour after tagging and exposure: The porpoises tagged in 2018 did not have time to return to feeding before exposure, but the four porpoises tagged in 2019 were feeding within 19–75 min after tagging. After AHD exposure, five porpoises were apparently feeding within 16–42 min, whereas the tag fell off the sixth porpoise shortly after exposure and before it had started feeding (Table 1). The tags recorded data for 2–44 h (Table 1), allowing us to estimate acute exposure effects (15-min exposure compared to 15-min prior to exposure).

### Startle and flight behaviour

**Figure 2:**
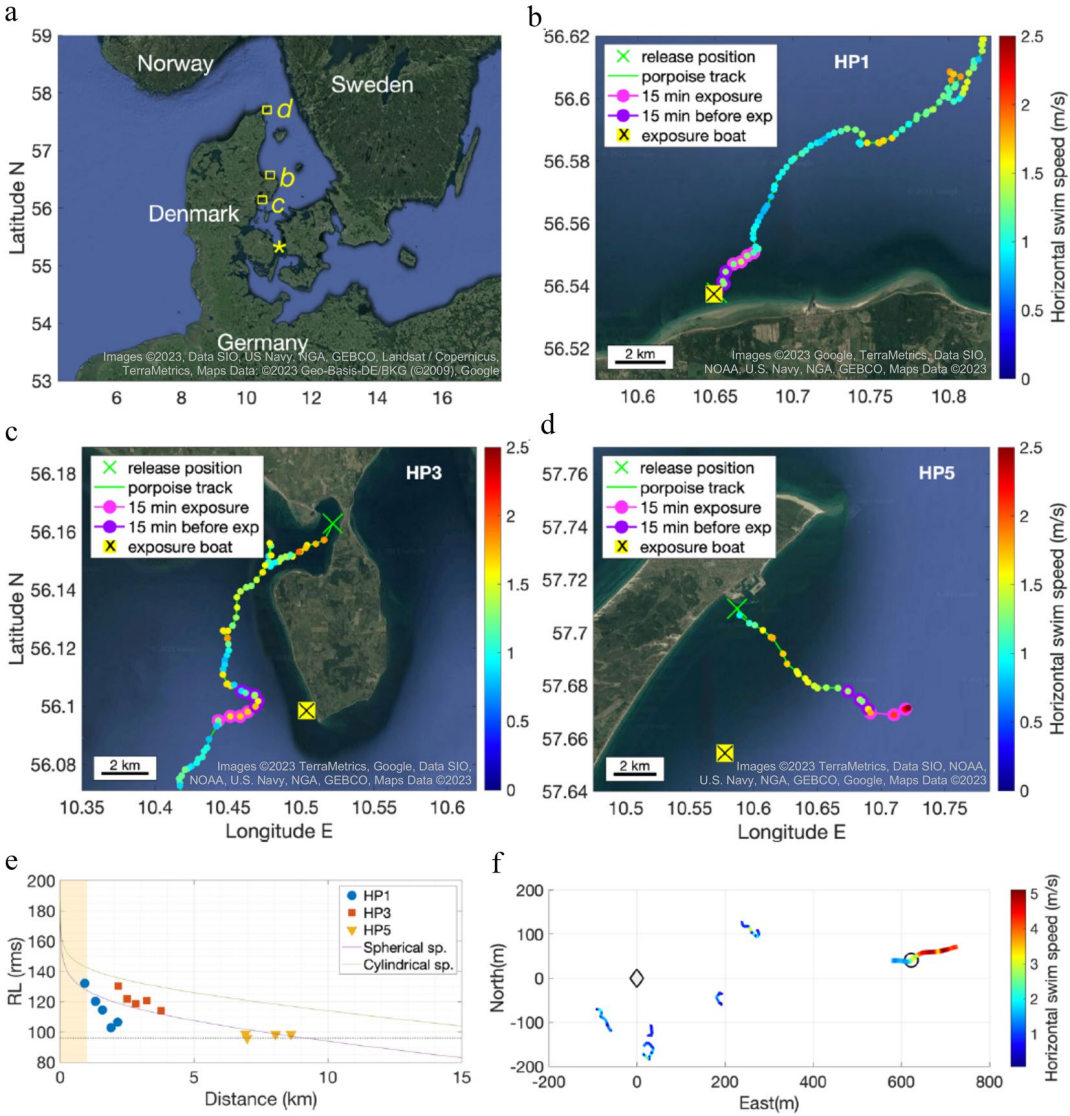
Exposure sites, GPS-tracks of AHD-exposed porpoises and measured sound exposure levels with distances. (**a**) Overview map of exposure locations for the six tagged porpoises in Danish waters. Squares mark the exposure locations of porpoises HP1, HP3 and HP5 (tagged together with HP6). The asterisk marks the location where HP2 and HP4 were tagged. The un-tagged porpoises, observed by drone, were exposed in Kerteminde Bay, close to the asterisk. (**b**) HP1 was exposed to the AHD (yellow box with black cross) 16 min after being released (green cross, under the yellow box here) from the tagging boat. GPS-positions are color-coded to show calculated horizontal travel speeds (running 5-sample means). The porpoise did not change direction significantly during exposure (pink track) compared to 15-min before exposure (purple track). (**c**) HP3 was exposed 202 min after release. At exposure start, HP3 changed direction with increased travel speed (pink track). (**d**) HP5 was exposed 100 min after release. Four minutes after exposure start, HP5 headed away from the sound source at high speed (pink track). The tag detached from the animal 4 min after the exposure stopped. (**a**–**d**) Satellite imagery is obtained from Google Maps using MatLab^72^. (**e**) Received levels (rms fast) of AHD pings are plotted as a function of distance between porpoises (with GPS-data) and the AHD device. From each GPS-position during the exposures, the 5 nearest pings in time were used to calculate a median RL at the distance. Two sound propagation models are plotted in green (cylindrical spreading) and purple lines (spherical spreading), with a medium sound absorption of 1.5 dB/km. The lowest initial RL, at which a porpoise increased MSA, was at 98 dB re 1 µPa rms (horizontal broken line). The AHD RL were > 20 dB higher than that at 1 km, which is the deterrence distance often desired to mitigate adverse effects from pile driving (yellow area). (**f**) For comparison with tagged animals, un-tagged porpoises were observed by drone and exposed to AHD-playback. Porpoise positions relative to playback-boat position (Diamond) are plotted for control trials (thin lines, n = 5) and one exposure trial (fat line, n = 1). The circle indicates the porpoise position at exposure start, where swimming speed increased with 150% (colour-coded). For all trials, 30 s prior to exposure and 30 s of exposure or “scam” exposure is depicted. The flight response of the untagged animal resembles that of the tagged animals.

**Figure 3:**
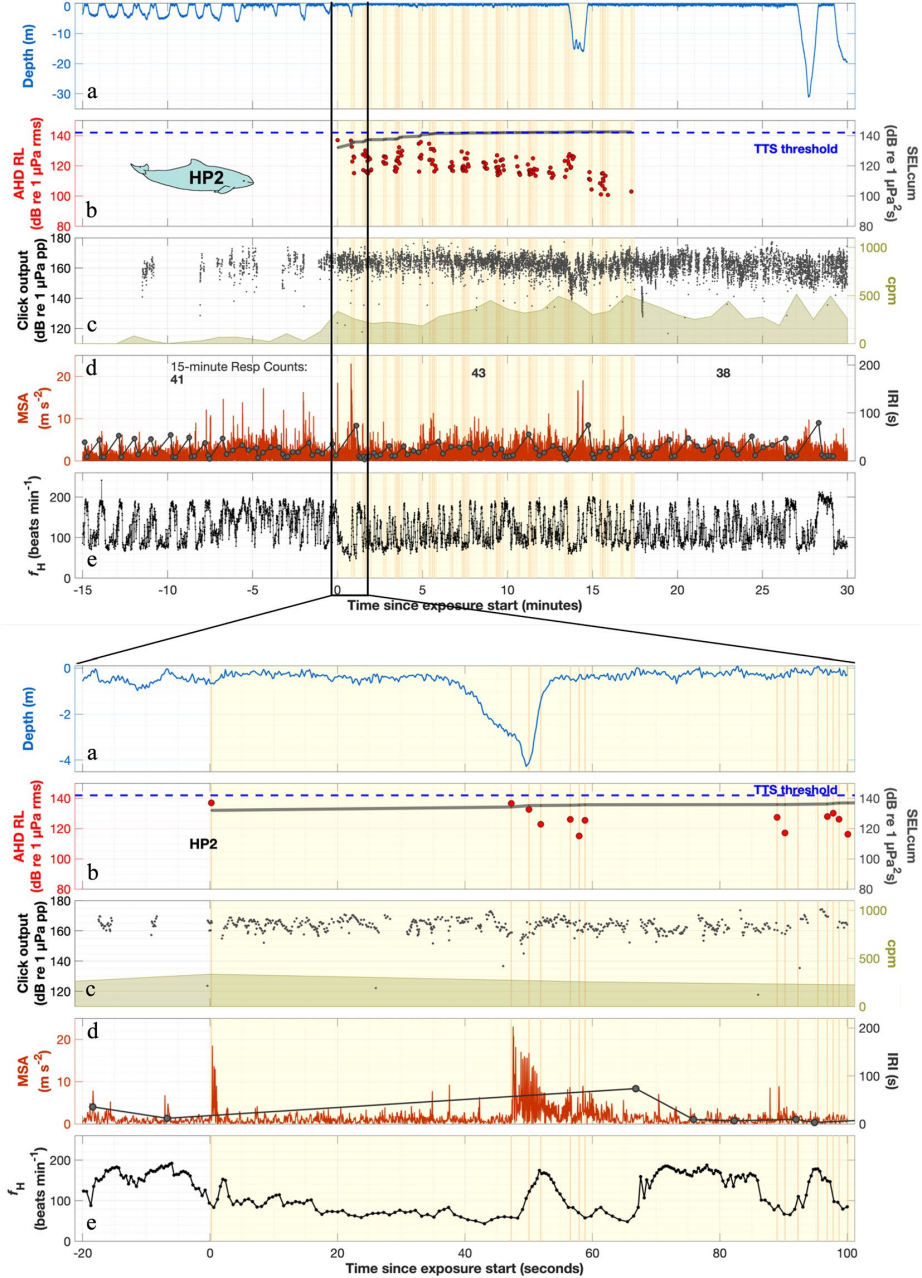
Behaviour around AHD exposure of HP2. 45 min of continuous data are presented: 15 min before exposure, 15-min exposure and 15-min after exposure. The exposure period (yellow patch) and each received AHD ping (orange vertical lines, and red dots in (**b**)) run through all the panels. (**a**) The dive profile (depth) shows that the porpoise swam near the surface for most of the 45 min. (**b**) During the exposure time, the AHD ping received levels (red dots) decreased from 132 to 82 dB re 1 µPa (rms fast) indicating that the animal swam away from the sound source. The cumulated Sound Exposure Level (SELcum) (dark grey line) increased with each ping, with the assumption of a non-leaky integration. The dashed blue line marks the threshold for temporary hearing threshold shift (TTS) of harbour porpoises for AHD single-pulse exposures, as determined by Schaffeld et al.^8^ for 20 kHz hearing. (**c**) Echolocation click rates (clicks per minute, cpm) increased during and after the exposure. (**d**) Minimum specific acceleration (MSA, red line) reflects strong transient fluking responses to the first pings (see zoom-in). Time intervals between respirations (IRI, dark grey line), i.e., breath-hold durations did not change. Each dot marks a breath. (**e**) Heart rate varied between 82 beats min^−1^ (10th percentile), when the porpoise was submerged, and 185 beats min^−1^ (90th percentile), when the porpoise was breathing. (zoomed in figure). After each of the first two bouts of exposure pings, the heart rate increased transiently to 153 and 174 beats min^−1^.

**Figure 4:**
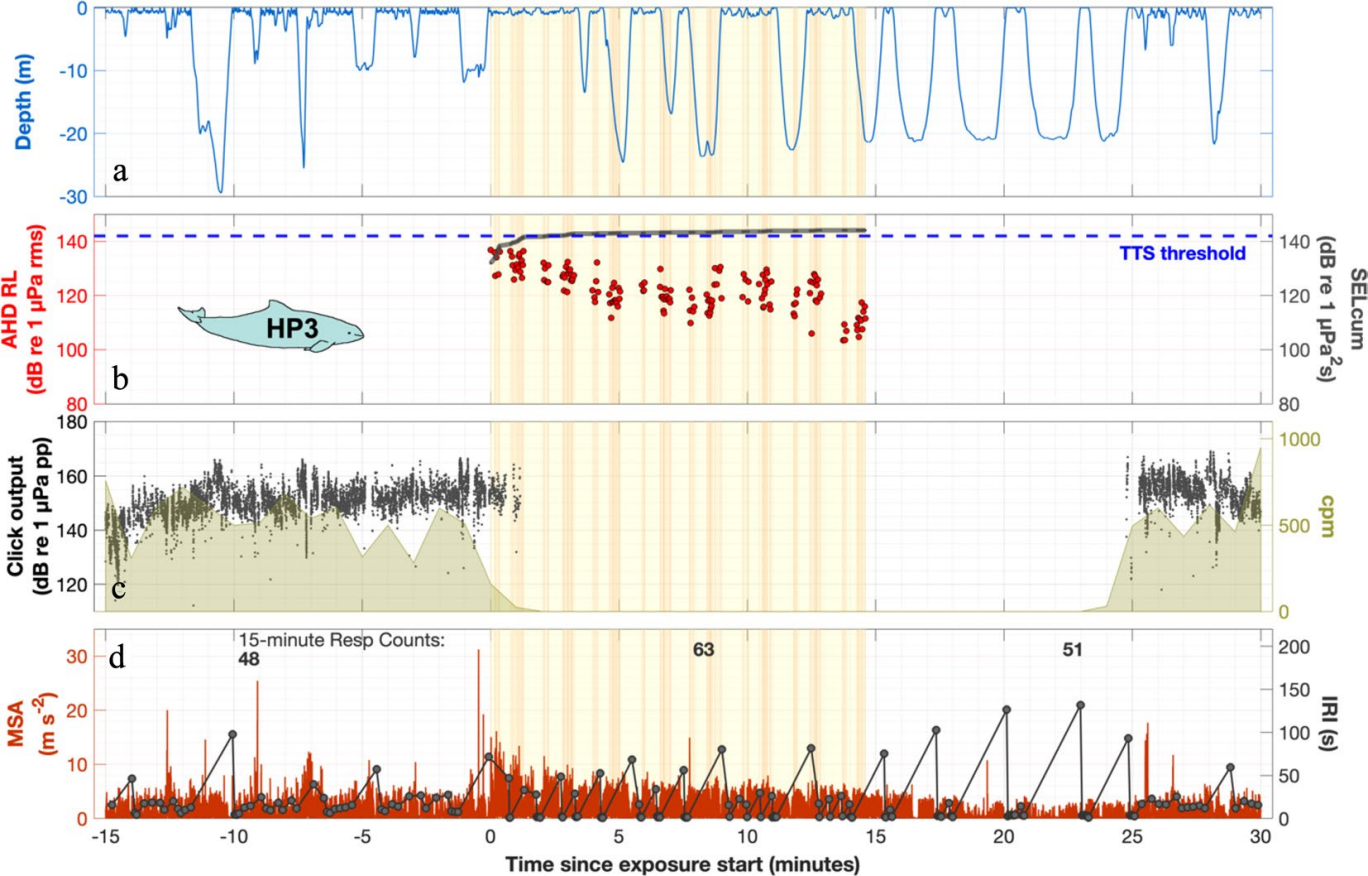
Behaviour around AHD exposure of HP3. See legend in Fig. 3 for general figure explanations. (**a**) The porpoise had surfacing periods right after the AHD exposure, where it seemed to lay at the surface several times. (**b**) The SELcum exceeded the TTS-threshold, indicating a risk of hearing impairment. Received level (rms fast) of the pings decreased as the porpoise swam away from the sound source. (**c**) Shortly after exposure start, the echolocation ceased (clicks per minute, cpm) and was not resumed until 10 min after the exposure had stopped. (**d**) Swimming effort increased, especially during the initial part of the exposure. During and after the exposure, the porpoise increased breath-hold intervals, but also increased number of breaths per surfacing, compared to before the exposure.

**Figure 5:**
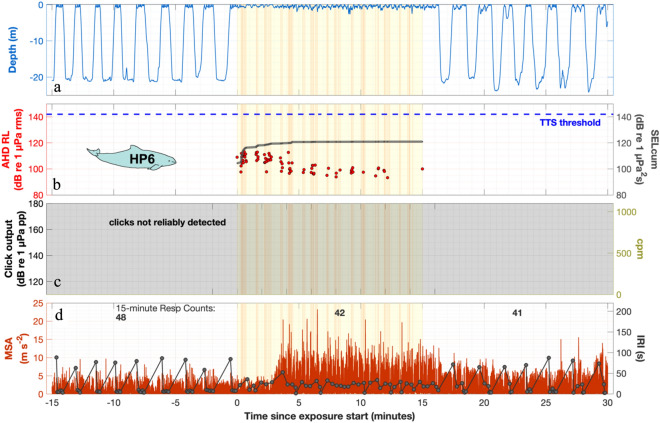
Behaviour around AHD exposure of HP6. See legend in Fig. 3 for general figure explanations. (**a**) This porpoise swam at the surface during the entire exposure period, but after about 1 min post exposure, it resumed to a dive pattern similar to the pre-exposure period. (**b**) This porpoise received moderate AHD levels (rms fast), and was not at risk of TTS. The decreasing received levels of pings indicates that the porpoise increased distance to the sound source. (**c**) The very high frequency echolocation clicks of the porpoise were not reliably detected because of the lower sampling rate of the audio recording on this specific mid-frequency dtag. (**d**) While remaining at the surface, swimming effort increased 3 min into the exposure period and was maintained at a high level for at least 15 min after the AHD exposure stopped. During the exposure, the porpoise was breathing at regular intervals.

The first response to the AHD exposure was an acoustic startle response in four of six harbour porpoises (HP1-HP3 and HP6, Fig. 1b). Five out of six animals (HP1-HP3, HP5-HP6)) increased distance to sound source as deduced from the decreasing AHD RL (verified by GPS data when available, Fig. 2b–d), while increasing swimming effort (95th percentiles of MSA over 5-s bins) with 58% on average (Figs. 3, 4, 5, and S1–S3, Tables 2, S1). Horizontal travel speeds during exposure increased 26% on average compared to 15 min before (GPS-data from HP1: 5% increase from 1.3 to 1.4 m s^−1^; HP3: 30% increase from 1.4 to 1.8 m s^−1^; HP5: 43% increase from 1.3 to 1.9 m s^−1^, Fig. 2b–d). The non-fleeing animal (HP4) made a deep U-shaped dive for the first two minutes of the exposure (Fig. S2) with a total 31% decrease in swimming effort (Tables 2, S1). All animals were predominantly in visual crypsis; during exposure, the porpoises were in crypsis on average 82% of the time (range 70–99%, Table S1), which was on average 15% (range – 2–33%, Table 2) more than before exposure (on average 67%, range 59–80%, Table S1).

**Table 2:** Response table for the six porpoises exposed to AHD.

Animal ID	Response Index (count of all responses irrespectable of direction)	Startle	Acute exposure effects (15-min exposure compared to 15 min before)						
			Crypsis			Escape behaviour			Hearing
		Startle response (jerk within 0.2 s of first ping)	Time in visual crypsis (% difference)^a^	Biosonar click rate (% change)	Biosonar output level (dB change)^b^	Swimming effort (% change in MSA)	Increased distance to AHD^c^	Resp. rate (% change)	Risk of TTS^d^
HP1	7	**Yes**	− 2	**− 39**	**− 8**	**17**	**Yes**	*− 11*	**Yes**
HP2	6	**Yes**	**33**	*690*	− 1	**25**	**Yes**	5	**Yes**
HP3	7	**Yes**	**8**	**− 98**	− 1	**32**	**Yes**	**31**	**Yes**
HP4	4	No	**10**	**− 27**	− 2	*− 31*	No	*− 15*	No
HP5	5	No	**15**	**− 53**	**− 8**^**e**^	**75**	**Yes**	4	No
HP6	5	**Yes**	**26**	^f^		**141**	**Yes**	*− 13*	No

Bold values indicate a behavioural change according to the hypothesized response indicated by the headlines, while italics values indicates an opposite change.

^a^Visual crypsis is defined as within 2 m from surface or apparent seafloor as evaluated from depth data and bathymetry.

^b^Calculated from 95 percentiles of all clicks in the respective 15-min periods.

^c^i.e. increased distance, as seen by decreased AHD RLs and in GPS data when available (HP1, HP3, HP5).

^d^Risk of effect on hearing ability, i.e. close to risk of TTS, according to TTS onset at 142 dB re 1 µPa^2^s for an AHD single pulse (Schaffeld et al.^8^).

^e^Only 10 min included in the exposure period in click level comparison, since tag slid at this time.

^f^The audio-recording on the mid-frequency tag (lower sampling rate) on HP6 was not suitable to detect and quantify porpoise high frequency clicks satisfactorily.

### Echolocation behaviour

During AHD exposure, four of the six porpoises (HP1, HP3-HP5) decreased their echolocation click rate by on average 54% (27–98%), and two of these (HP1, HP5) additionally decreased apparent click output level (95th percentile) by 8 dB (Table 2). One porpoise (HP2) increased echolocation rate by 690% during the exposure (Tables 2, S1). Importantly, the porpoises responded in different ways to the AHD exposure: HP1 instantly reduced click output level, and overall click rate during the exposure interval (Fig. S1); HP2 increased click output levels within seconds (Fig. 3); HP3 altered echolocation behaviour a few minutes into the exposure, but then seized echolocation completely for 20 min (Fig. 4); HP4 decreased click rates moderately at first during the exposure, but then resumed clicking toward the end of the exposure (Fig. S2); HP5 decreased click output level instantly, while click rate decreased more gradually (Fig. S3); The tag on HP6 did not sample fast enough to allow for reliable click detection.

### Heart and breathing rate responses

The two animals with heart rate recorders (HP2, HP4) had divergent responses to the exposure both regarding startling and heart rate changes. HP2 startled upon exposure to the high initial RL (137 dB re 1 µPa, rms-fast of first ping, or mean 131 dB re 1 µPa rms-fast of five first pings). HP2 then had transient, but dramatic increases in heart rate (i.e. tachycardia) from 83 to 153 beat min^−1^ (84% increase) and 57 to 174 beats min^−1^ (204% increase), while breath-holding and diving (Fig. 3, zoom-in). The other porpoise (HP4) was exposed to much lower RL (102 dB re 1 µPa first ping or mean 99 dB re 1 µPa rms-fast of five first pings), and gradually decreased diving heart rate (50-s median), from 69 to 51 beats min^−1^ (minimum instantaneous heart rate was 39 beats min^−1^), as it dove toward the presumed seafloor in a for porpoises unusually long 3-min breath-hold dive (Fig. S2).

The respiration rate was unchanged in two animals, while the four others changed respiration in different ways during exposure compared to the 15 min before exposure, ranging from a 15% decrease (HP4, from 3.9 to 3.3 min^−1^) to 31% increase (HP3, from 3.2 to 4.2 min^−1^) (Tables 2, S1).

### Received level and effect distances

By plotting AHD RL from GPS-DTAGs with corresponding ranges from the porpoise to the exposure boat of three different exposure events (HP1, HP3, HP5), we found that the sound propagation largely followed a spherical spreading model for the distances and areas considered here (Fig. 2e).

### Verification of flight response without tags

Using a drone-mounted camera, un-tagged porpoises were observed during five control trials for on average 308 s (min–max: 70–533 s) and during one exposure trial lasting 371 s (baseline: 85 s; exposure: 286 s). In the successful exposure trial, a group of seven porpoises (five adults and two calves) were exposed, where the focal animal was an adult with a calf (Suppl. video SV1). All animals reacted by fast swimming in different directions generally away from the sound source. One mother-calf pair initially swam in different directions, while the focal porpoise and calf where swimming in close coherency during the exposure. The focal porpoise showed a similar avoidance behaviour as the tagged porpoises, i.e. swimming away from the sound source at more than doubled swimming speed (4 m s^−1^ average the first 30 s of exposure, compared to 1.6 m s^−1^ average during the preceding 30 s) (Fig. 2f). The initial RL was 119 dB re 1 µPa (rms-fast). The control playbacks did not evoke apparent avoidance of the sound source or fleeing, in that the porpoises did not swim away from the exposure boat (Fig. 2f) and average swimming speed varied between 0.7 and 1.1 m s^−1^. For control trials, the median swim velocity before and during 'mock exposure' was not significantly different (Wilcoxon signed rank test: − 0.22, 95% Confidence Interval (CI) − 0.80 to 0.61, p = 0.438). In the exposure trial, swim velocity increased on average by 2.46 m/s. This increase is four times the upper range of the 95% CI of the control trials, underlining that the porpoise clearly increased swim velocity when exposed to AHD. Furthermore, in the control trials, we observed no signs of distress or annoyance to boat or drone presence, such as porpoising, chuffing, or fleeing.

## Discussion

Porpoises seem to respond to AHDs at ranges far beyond the usual mitigation goal of 1 km and at moderate estimated RLs^7,9,12^. For example, Brandt et al. detected a significant decrease in echolocation activity out to a distance of 7.5 km (corresponding to an estimated RL of ~ 100 dB re 1 µPa rms) during AHD exposure^6^, while Dähne et al. reported decreased click activity out to 12 km from a Lofitech AHD (corresponding to an estimated RL of ~ 90 dB re 1 µPa based on spherical spreading^50^ and 1.5 dB/km absorption)^51^. It is unclear if such reduced click detections are a result of displaced animals or less clicking by animals staying in the area. Importantly, the details of the physiological and behavioural responses to actual received sound levels is unknown in these PAM studies. By tagging six harbour porpoises with multi-sensor DTAGs, we here sought to provide more details of behavioural and physiological responses of individual harbour porpoises to measured received levels from AHDs to address the sensitivity of porpoises to these common marine sound sources.

### Flight, orienting and crypsis responses

Five porpoises displayed apparent flight responses with increased swimming effort and increased distance to the sound source. The three porpoises with GPS tracks (HP1, 3, 5) headed away from the sound source with a 15-min mean horizontal travel speed of 1.4, 1.8 and 1.9 m s^−1^ during the exposure. These speeds are comparable to speeds of 1.3–3.2 m s^−1^ during previous AHD exposure^9^ and 2.2 m s^−1^ of wild porpoises exposed to startling pulses^19^. Importantly, they are well above the average travel speed of 0.65 m s^−1^ (0.15–2.8 m s^−1^) estimated from successive GPS locations of un-exposed harbour porpoises in the same area^52^. Of the five apparently fleeing porpoises, four that had startled initially (HP1-HP3, HP6) fled from the AHD immediately with strong fluking (Figs. 3, 4, 5, and S1). Meanwhile HP5 did not take flight initially; however, four minutes after receiving the first ping, it started swimming near the surface with increased swimming effort (Fig. S3). Feeding buzzes and depth data suggest that HP5 had just found a patch of prey, which could have been a strong incentive to ignore the low RL at first.

HP4 did not flee at all, but instead seemed to rest near the seafloor quietly, which could be an orienting response to the novel stimuli^53^ (Fig. S2). Narwhals have been observed to display freeze-responses (causing them to slowly sink) in response to ship noise^17^. Furthermore, a similar quiet descent has previously been reported in a porpoise exposed to a passing fast ferry^45^, and porpoises, exposed to a pinger, had responses varying from extremely rapid deterrence to no reaction at all^54^. Thus, porpoises may respond to exposures both with flight (predominantly in our dataset), but also with freeze-like orienting responses.

All of the porpoises spent the majority of the time during AHD exposure at close proximity to the surface or the seafloor and more so than before exposure. This apparent avoidance of the open water column may be a mean to achieve visual crypsis as an antipredator response to the AHD.

### Echolocation behaviour and detection implications

We predicted that flight could entail either increased echolocation to increase sensing while swimming at high speeds, or decreased echolocation to achieve acoustic crypsis from perceived predators. Four porpoises (three of which were fleeing) decreased echolocation click rate by on average 54% (27–98%), and furthermore, two of those porpoises reduced the 95th percentile click output level by 8 dB (Table 2).

These responses are consistent with described anti-predator responses of other species of toothed whales, such as beaked whales^18^. The risk-disturbance hypothesis^15^ involves that an anthropogenic noise, such as a sonar, may resemble vocalizations of predators, and therefore trigger innate responses evolved in a prey-species to increase survival under specific predatory pressures such as from killer whales or other large delphinids. The innate response characteristics and triggering stimuli will under this scheme depend on the biology, general predation-risk and anti-predator responses of the exposed species. For species such as beaked whales, narwhals and harbour porpoises, visual and acoustic crypsis help to achieve one major goal: not to be detected and later harassed, attacked or killed during an escape. However, these acutely life-saving responses also come with acute increased risks. When fleeing from one perceived predator, the resulting diversion of attention increases the risk of predation by another predator, and perhaps also lead to other fatal mishaps: If our results are applicable to porpoises exposed to AHDs in general, we speculate that increased swimming speed along with decreased bio-sonar output result in decreased detection ranges, vigilance and response time, which in turn may increase the risk of being bycaught in fishing gillnetsIn support of this, signs of entanglement has been observed in porpoises that stranded following a naval exercise^55^. Furthermore, the ability to detect net echoes may be impaired by potential AHD-caused TTS, resulting in a decreased functional detection range. Hence, while the vast majority of porpoises alarmed by AHDs, or mid-frequency active sonars, will flee without encountering a gillnet, even the small percentage that does could perhaps offer a partial explanation for why so many porpoises get bycaught despite their capability to detect and avoid nets at relatively long ranges^56^.

Interestingly, the reduced biosonar output shown here suggests that the decrease in echolocation detections in PAM studies may not only be caused by displacement, but also stem from lower click intensities and rates during exposure to AHD noises; fewer click detections hence does not necessarily mean fewer animals in an area^57^.

### Physiological responses

Very little is known about how wild marine mammals respond physiologically to acoustic stressors. We hypothesised that porpoises would employ flight responses, like terrestrial mammals, with increased respiration rates, heart rate and muscle perfusion to support oxygen delivery^58^.

Five fleeing porpoises (HP1-HP3, HP5-HP6) increased their swimming effort with on average 58% (range 17% to 141%) upon exposure. The inconsistent effect on respiration rates (from 13% decrease to 31% increase, Tables 2, S1) may be due to some of the animals deferring the needed ventilation until after escaping while relying on increased anaerobic metabolism, or they might also adjust their lung tidal volume to accommodate the increased oxygen needs during flight behaviour^59,60^. Even though we cannot speak to energetic costs of the flight, an escape is expected to be more costly than normal locomotion as found for both marine and terrestrial predators in a recent comparative study^61^. For populations that have impaired health status, due to for example lung parasite loads^25^, the capacity for flight may be affected and the time needed to recover could be longer.

The two porpoises with heart rate recordings responded in different ways. In one porpoise (HP4), the diving heart rate decelerated during exposure, as it quietly dove (Fig. S2), which could be considered a defensive freeze or orienting response^53^. Cardiac freezes, i.e. extremely low heart rates, have been observed in narwhals fleeing from a capture site; a response suggested to conserve blood oxygen and prolong dive times while the narwhals were escaping in crypsis^62^. In harbour porpoises in captivity, heart rates as low as 10 beats min^−1^ have been observed^39^, so the reduction to 51 beats min^−1^ of HP4 does not qualify as a cardiac freeze.

HP2, which was startling, had several dramatic transient accelerations of diving heart rate (Fig. 3, insert). Even though HP2 was still breath-holding, the tachycardia was as high as when breathing, where blood oxygen can be expended unrestrictedly. For captive diving porpoises, it was previously found that the acoustic startle response was decoupled from autonomic heart rate changes^22^, but it is known from terrestrial animals that emotional state and fear affects likelihood and amplitude of startling^63^. Thus, perhaps the amplitude and likelihood of startle-related heart rate change is increased in this wild harbour porpoise that had recently been tagged. Alternatively, or additionally, the heart rate increase may be a consequence of muscular vasodilation from increased swimming effort, and as such in line with classic exercise physiology and studies on captive bottlenose dolphins and porpoises^39,49^. Wild hunting porpoises do not seem to alter diving heart rate consistently with observed exercise levels^64^; but in a context of elevated stress, an increased sympathetic (or alleviated parasympathetic) tone could perhaps enhance cardiovascular responses to exercise. An increase in heart rate at this scale is driven by a significantly increased peripheral perfusion, which in a breath-holding animal would quickly lead to deoxygenation of the blood^65^. If such responses, albeit transient, occur in any deeper-diving individual, it would not only have to cut its dive short to surface for oxygen; the response could also lead to increased nitrogen flux to tissues at depths before lung collapse. If recurring, this may put the animal at increased risk of decompression sickness^30,31^. Many of these considerations on the physiological responses to noise in marine mammals remain speculative at this stage owing to the difficulty with which they can be collected in the wild. The only example, to our knowledge, of detailed data from wild individuals exposed to a noise stressor is from narwhals exposed to airguns. During exposure, the narwhals had lowered dive heart rates (including intense bradycardia) while their exercise level was increased compared to no-exposure^66^. The different heart rate response in our study highlights the need to better understand how behaviour and physiology interplay in wild marine mammals as a function of natural and disturbed conditions.

### Tagging effects

To provide important information of the fine-scale individual behavioural and physiological responses to AHDs, we exposed porpoises that had recently been bycaught, shortly handled and tagged with suction cup archival sound and movement tags. The goal for a tagging study is to achieve and use data that are un- or minimally affected by the tagging to represent the investigated behaviour and physiology as naturally as possible.

In our study, we use foraging behaviour (feeding buzzes) as an indicator that the porpoises are returning to approximate normality. The porpoises HP1-HP2 were exposed so shortly after release that they did not resume feeding before exposure (Table 1) and could still be acutely affected by the tagging procedure. HP3-HP6 started feeding 19–75 min (0.3–1.25 h) after tagging, suggesting that the immediate tagging effect was wearing off relatively quickly, in accordance with a previous study, which reported that porpoises were feeding 0.1–4.1 h after tagging using the same general protocol^45^. After exposure, feeding was resumed 16–42 min after the end of the exposure, indicating a similar recovery time, and not potentiated as might have been the case if the porpoises were strongly sensitised from tagging. Even after the resumption of feeding, the porpoises could potentially still be affected, even though this has never been investigated for this exact tagging protocol. Considering how cardiac responses are affected by emotional state^63^, it is possible that the recent tagging affected cardiac responses in the two animals that were exposed 16–17 min after tagging (HP1, HP2).

To explore if our results were heavily biased by tagging effects, we also exposed un-tagged porpoises monitored with a drone-mounted camera. Here, in the one successful exposure trial, a small group of un-tagged porpoises responded similarly to the tagged porpoises (Fig. 2f, Suppl. Video SV1). The focal porpoise swam away from the sound source at travel speeds (2.5 m s^−1^ during the 5-min period) comparable to or higher than those during exposure from the tagging study (1.4–2.5 m s^−1^, Fig. 2a–d). This suggests that the flight behaviour in response to AHDs is not a result of tagging-induced sensitisation. The focal porpoise had a calf and its sensitivity to disturbance might thus have been increased. On the other hand, in a study where drone-observed porpoises were exposed to pingers, an adult with a calf had similar responses and swimming speeds to a single adult porpoise^54^. What is evident from both exposure of tagged and untagged animals is that every individual responded to the AHD. Moreover, these findings are overall in accordance with observations of porpoise deterrence due to AHDs^5,6^.

Deterrence distance, habituation and management implicationsWe find that harbour porpoises react at distances up to more than 7 km (RL: > 98 dB re 1 μPa, rms-fast) from the AHD (Tables 1 and 2), which is consistent with the 7.5 to 12 km range with decreased click numbers/events of PAM studies^6,51^. The RL of response-inducing exposure is also comparable to a study of Mikkelsen et al. (2017), where the majority of observed porpoises avoided a simulated AHD signal (12 kHz with SL ~ 162 dB re 1 µPa rms-fast @ 1 m) within a distance of 525 m corresponding to a RL of 98 dB re 1 μPa (rms-fast)^7^.

While deterrence devices need to be effective to avoid entanglement in fishing nets and TTS/PTS from construction work, the risk and effect caused by the deterrence should not exceed the risk and effect of the activity the animals are deterred from. Therefore, the SL of deterrence devices is an important parameter to consider in light of the received level that trigger a desired response. All of the further risks arising from the anti-predator response of a porpoise (attention diversion, reduced bio-sonar output during escapes, and potential physiological consequences) are risks necessary to take by an animal escaping a predator. However, in the benign case of using AHDs to deter porpoises to 1 km distance from a construction site, these risks may exceed that of the short-range (< 1 km) construction-imposed risk of hearing damage, especially considering the large range of the described AHD effects (at least 7 km). The lowest initial AHD RL in this study (96 dB re 1 μPa, rms-fast, HP5) evoked an apparent flight response, while acoustic startle responses were evoked with initial RL as low as 106 dB re 1 μPa, rms-fast (HP6). This matches the observations made by Tougaard et al., who predicted behavioural response thresholds based on the sensation level of the signals of around 45 dB above the pure tone hearing threshold^11^, or equal to about 100 dB re 1 μPa at 14 kHz.^10^. The observed responsiveness at levels 90 dB below SL results in very large effect ranges (> 7 km from a 190 dB re 1 µPa @ 1 m source level AHD, Fig. 2e)^50^. Hence, an AHD deployment could lead to large-scale habitat exclusion and quality degradation of porpoises over an area covering much more than 100 km^2^.

The problem of a much larger impact zone than desired not only applies to mitigation zones around offshore construction, which are growing due to renewable energy expansion targets, but also to areas where AHDs are used to deter seals from fish farms. Findlay et al.^67^ demonstrated how the increasing use of AHDs in fish farms in Scottish waters from 2006 to 2016 have resulted in a substantial geographic area where AHDs are a source of chronic underwater noise pollution. When used over large areas and extended periods, AHDs may have substantial impacts on target and non-target species. Yet, the long-term effectiveness of preventing pinniped depredation has not been shown conclusively^3^. The widespread use, in concert with the responses demonstrated here, highlight the need to understand whether porpoises undergo a large-scale habituation or are suffering from large-scale habitat exclusion or degradation. Although the persistence of effects or potential habituation is not addressed, our data suggest that the responses do not stop at the end of the AHD exposure (Figs. 2, 3, 4, 5 and S1–S3). In a study, where wild porpoises were deterred from 15 min of seal scarer-like startling pulses, the same or new individuals reappeared at the study site ~ 31 min after the exposure stopped, with observation rates similar to pre-exposure after ~ 45 min^19^. This suggests a response beyond the exposure period, but not extending to permanent habitat exclusion. From captive studies, it appears that harbour porpoises can habituate to some degree to noise^22,28^, but persisting responsiveness is also reported^68^. Animals in captivity may, however, not be representative of wild conspecifics since they cannot flee and may be trained in noisy conditions. Wild harbour porpoises may be desensitized to pile driving over time^69^, but in areas of intense ship traffic, porpoises still show disturbance responses at moderate received levels^45^. Therefore, the presence of porpoises in an exposure area does not imply that they do not respond and pay a cost. Further studies are needed to gain insight on potential habituation or sensitization to AHDs, and long-term consequences of chronic exposures to AHDs.

Temporary or permanent hearing impairment are other potential effects of loud exposures. Schaffeld et al.^8^ have shown that a single AHD-pulse can inflict temporary hearing threshold shift (TTS) in porpoises (determined at 20 kHz, half an octave above the AHD) at sound exposure level (SEL) of 142 dB re 1 µPa^2^s^8^. When considering the cumulative sound energy shortly after exposure start, even at 2.2 km distance (HP3, Figs. 2c and 5), some of our exposed animals get close to that TTS limit assuming full cumulative effect over the exposure duration. Thus, not only do AHDs with high SLs scare porpoises out to substantial distances, but their hearing may also be impacted temporarily out to several kilometres. We therefore agree with Schaffeld et al.^8^ that current source levels of AHDs are too high when used for porpoises given the large off-set between levels that evoke behavioural responses and those causing TTS. Todd et al.^70^ modelled acoustic deterrent device sound propagation and calculated TTS-risk for a number of marine mammal auditory groups following Southall et al.^71^, and found that very-high frequency cetaceans, such as harbour porpoises, may be at risk of TTS at ranges of 4–31 km, where SL was the most important variable for the impact^70^. In our study area, even a moderate decrease of AHD source level by 10 dB will significantly reduce the distance with risk of potential hearing impact (from 0.9 to 0.3 km in the area of HP1, or from 2.2 to 0.6 km in the area of HP3 when estimating the transmission loss by TL = 20 × log(R) and using the SL of our AHD. Hence, the area where the AHD potentially inflicts risk of hearing impairment would be reduced by a factor of 9 (A = π × r^2^), thus potentially affecting ~ 90% less porpoises, assuming an even distribution of animals. This potential efficacy of reduced SLs has recently been demonstrated by Hiley et al.^19^, who exposed wild harbour porpoises to 15 min of 10.5 kHz startling noise pulses at a SL of 176 dB re 1 µPa @ 1 m, i.e. ~ 10 dB lower than many commercial AHDs. They still saw deterrence out to ~ 2 km and complete exclusion out to ~ 1 km^19^. The effective deterrence of porpoises was therefore achieved while greatly reducing the risk of TTS from the deterrence pulse or inducing unnecessary displacement of animals at long ranges.

If porpoises consistently swim away from an AHD at a received level > 100 dB re 1 μPa (rms), the SL of an AHD might even be reduced by 26 dB from 190 to 164 dB re 1 µPa (rms) @ 1 m and still deter animals out to a 1-km radius from a construction site (Fig. 2e): A transmission loss of 62 dB over 1000 m leads to a RL of around 102 dB re 1 µPa. A SL reduction to 164 dB re 1 µPa (rms) @ 1 m will effectively eliminate the risk of TTS, because TTS would only occur at very short ranges from the device. Furthermore, the number of porpoises affected behaviourally and physiologically by the AHD would on average be reduced to < 5% compared to a SL of 190 dB re 1 µPa (rms) @ 1 m. If the objective of the AHD is deterrence out to 1 km to avoid hearing impairment, current AHD source levels are considerably higher than necessary.

## Conclusion

All six tagged harbour porpoises responded to the AHD exposure at initial distances up to ~ 7 km, and at initial received levels down to 98 dB re 1 µPa (rms). A combination of increased swimming effort and speed with decreased echolocation output could increase the risk of entanglement in fishing gear. The changed echolocation behaviour may affect the accuracy of passive acoustic monitoring during exposures. Our data strongly support previous study conclusions that porpoises are deterred out to more than 7 km from an AHD, while the aim is often to deter out to 1 km, meaning that ~ 50 times more animals than desired are affected. As such we posit that the current AHDs manufactured to keep seals away from fish farms are too powerful to be used as a tool for mitigating hearing damage of porpoises around pile driving activities and that the extensive use of high SL AHD in widespread aquaculture areas can have substantial effects on porpoises unless they display widespread habituation. We therefore recommend substantial SL reduction of at least 20 dB of AHDs used as deterrence devices for porpoises, and possibly also other toothed whales.

### Supplementary Information

Supplementary Information.

Supplementary Video 1.

## Data Availability

Relevant data are within the paper and its Supplemental material.
