# Supplementary material for: Wild harbour porpoises startle and flee at low received levels from acoustic harassment device
Source: Sci Rep. 2023 Oct 4;13:16691. doi: 10.1038/s41598-023-43453-8 (PMC10550999; doi:10.1038/s41598-023-43453-8)
Supplement: Supplementary file 1 — Supplementary Information. [file 41598_2023_43453_MOESM1_ESM.docx]

**Supplemental figures and video**

Wild harbour porpoises startle and flee at low received levels from acoustic harassment device

Siri L. Elmegaard, Jonas Teilmann, Laia Rojano-Doñate, Dennis Brennecke, Lonnie Mikkelsen, Jeppe D. Balle, Ulrich Gosewinkel, Line A. Kyhn, Pernille Tønnesen, Magnus Wahlberg, Andreas Ruser, Ursula Siebert, Peter Teglberg Madsen

**
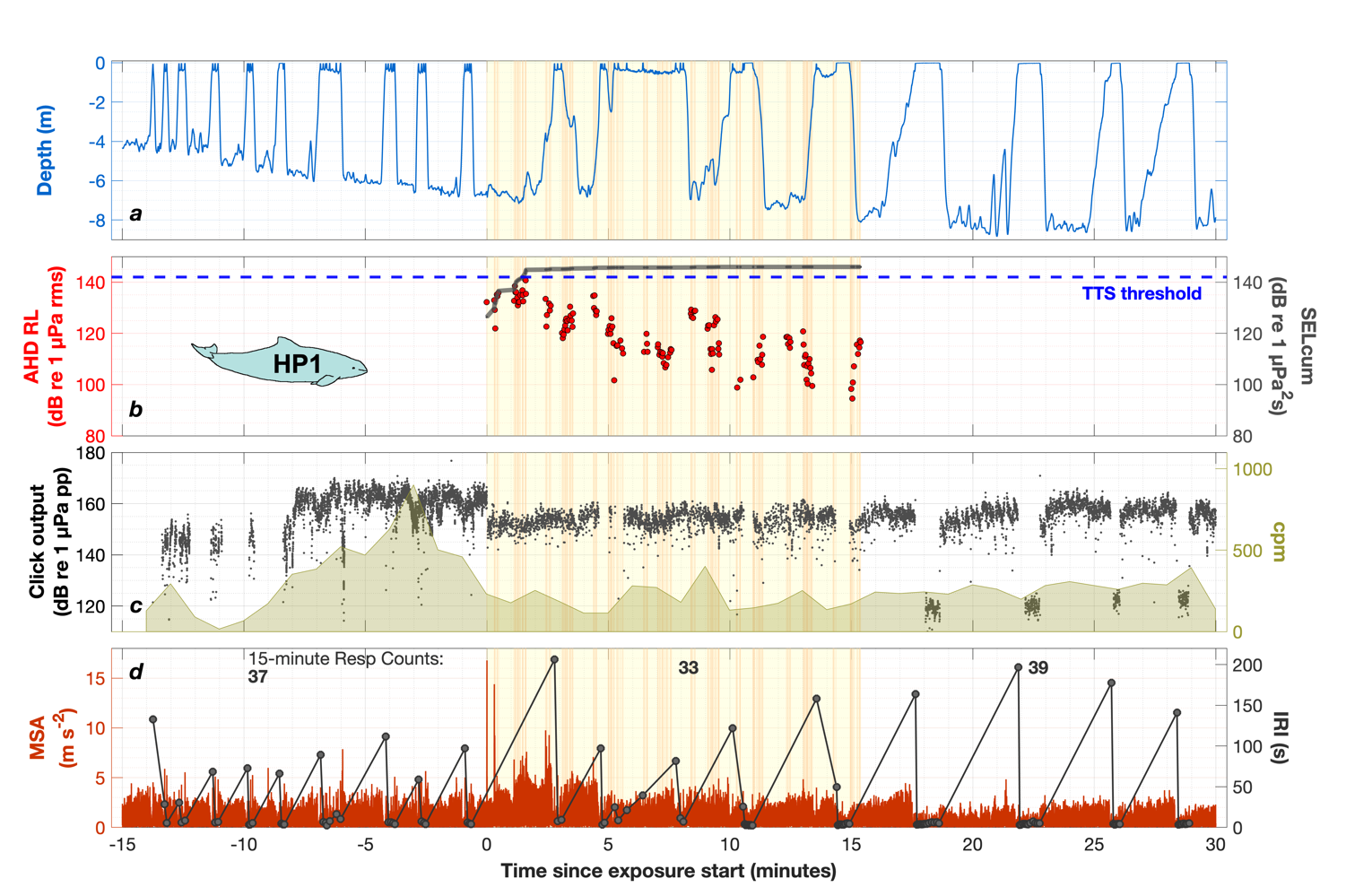
**

**Figure S1.** **Behaviour around AHD exposure of HP1**. See legend in Figure 3 for general figure explanations. (a) The animal was diving to 6-8 m depth while holding its breath for an unusually long 3.5 min as the exposure started. (b) The AHD received level was decreasing as the porpoise swam away from the sound source. After relatively few pings, the SEL surpasses the TTS threshold, indicating that the animal was at high risk of temporary hearing impairment. (c) Echolocation click intensities (click output) decreased by 10 dB as the exposure started. Clicks per minute (cpm) also decreased at exposure start. (d) The increased MSA, especially during initial exposure, indicates that the animal was swimming at increased effort and likely speed. This increased activity was linked with longer breath-holds (IRIs), from ~60 s before exposure to 100-200 s during and after exposure. After exposure, the coherent time at the surface and the number of breaths per surface period increased, while activity was low.


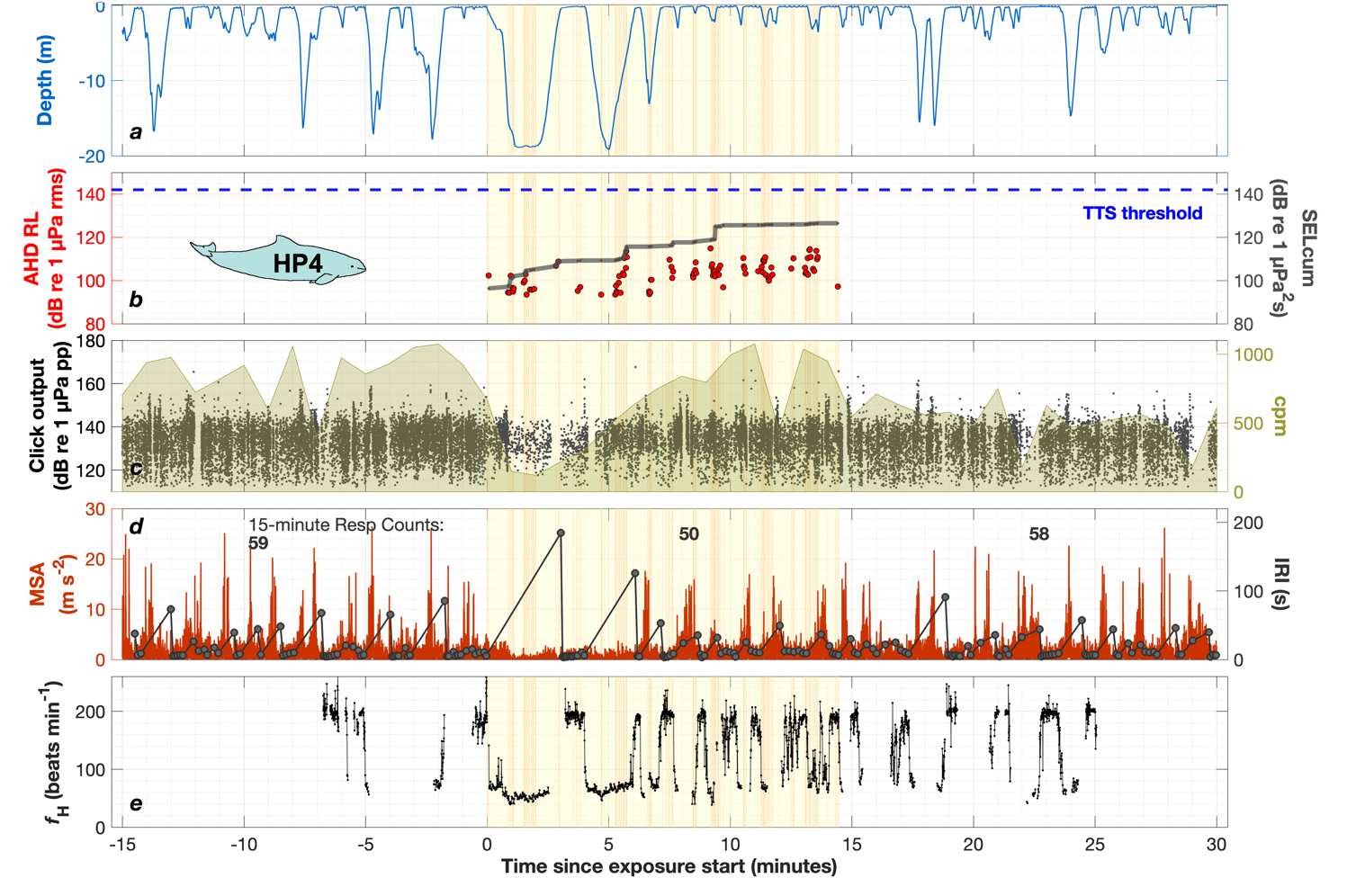


**
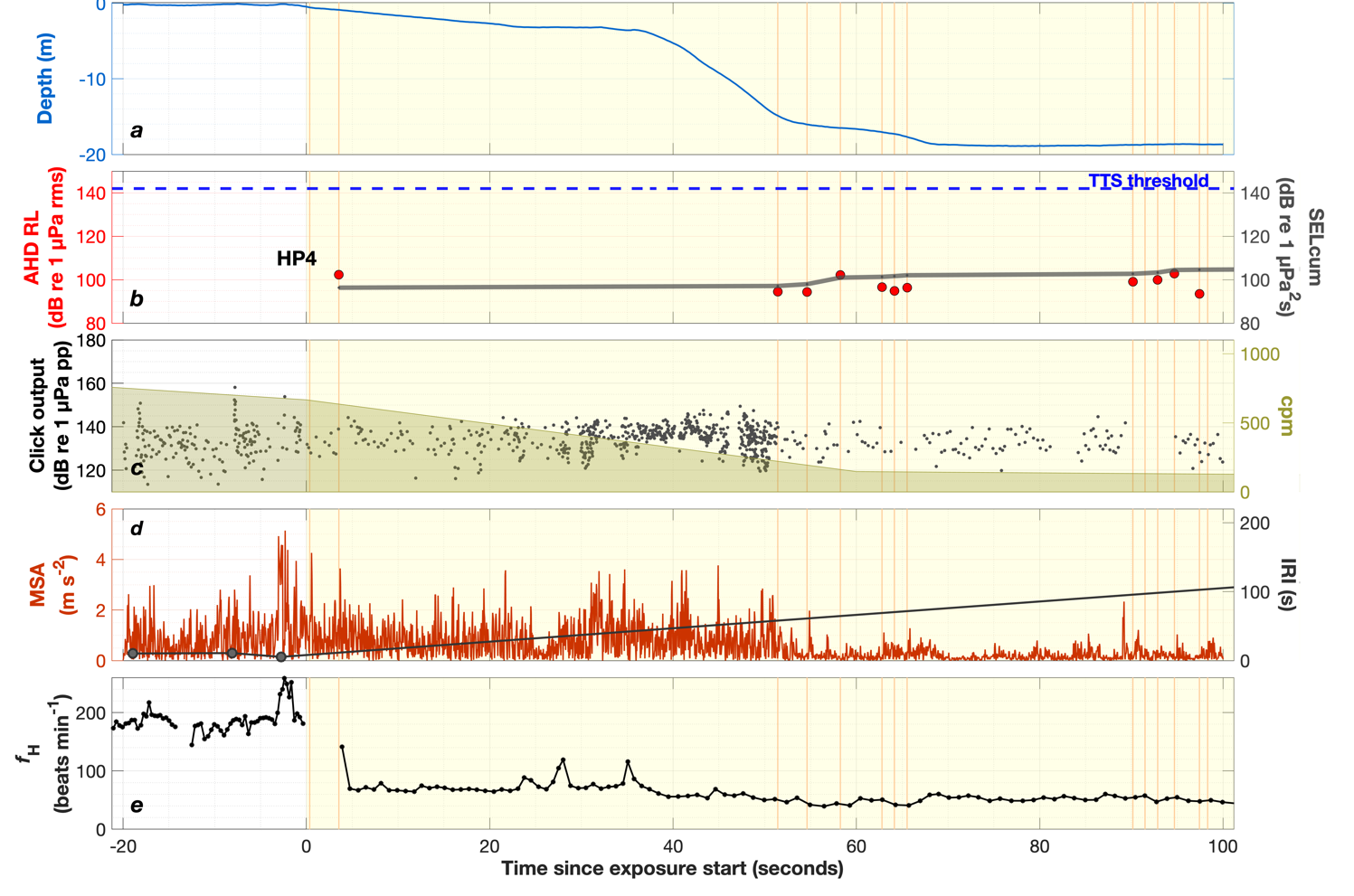
**

**Figure S2. Behaviour around AHD exposure of HP4**. See legend in Figure 3 for general figure explanations. (a) This porpoise performed a deep U-shape dive, as exposure started. (b) AHD received levels (rms fast) were moderate, which is also reflected in the SELcum, which was well below the TTS threshold. The slightly increasing AHD received levels suggesting that this porpoise did not swim away from the exposure site. (c) As the animal swam towards the seafloor, echolocation almost ceased for a few minutes (clicks per minute, cpm), but was back to pre-exposure level and rate after 5-7 min. (d) Movement acceleration was low, particularly after the first minute of exposure. Inter-respiration intervals show that the porpoise was breath-holding for two extended periods initially in the exposure period. (e) Heart rate varied between ~63 beats min^-1^ (10th percentile) when the porpoise was submerged, and 200 beats min^-1^ (90^th^ percentile), when the porpoise was breathing. During the 200-s breath-hold following the first AHD ping, the instantaneous heart rate fell as low as 39 beats min^-1^. No acute heart rate increases were observed in this animal at received AHD pings.


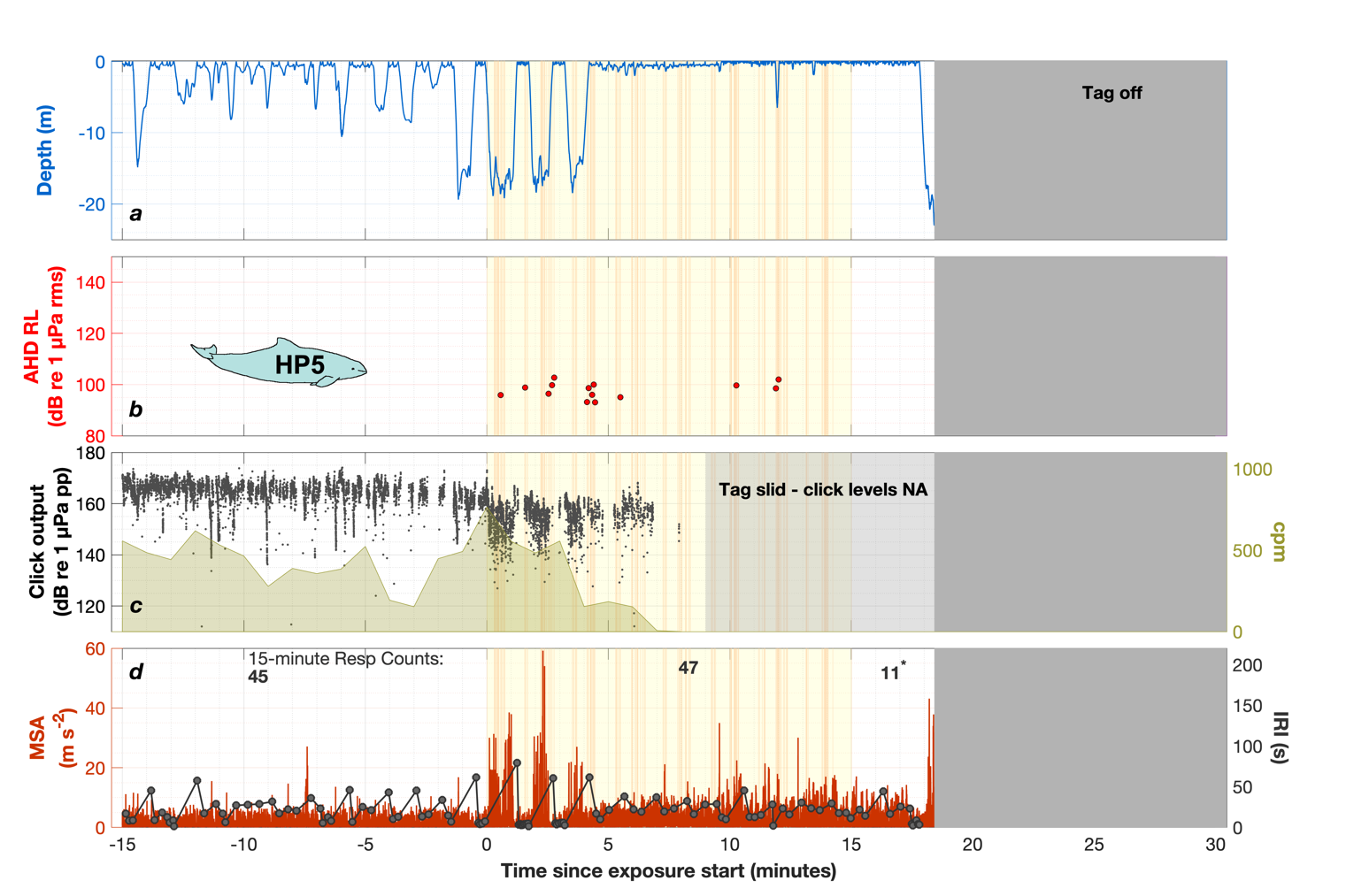


**Figure S3**. **Behaviour around AHD exposure of HP5**. See legend in Figure 3 for general figure explanations. (a) Approximately 5 min into the exposure, the porpoise altered dive behaviour staying close to the surface. 4 min after the exposure period ended, the tag detached at the bottom of the last dive. (b) The received levels (rms fast) were moderate. SELcum is not evaluated, since only a few pings had sufficient Signal-to-noise ratio to quantify RL. (c) The porpoise continued to echolocate with moderately high rates (clicks per minute, cpm) as the exposure started. This was linked with many feeding buzzes. Echolocation rates decreased as the porpoise swam close to the surface. 10 min after exposure start, the suction cup tag slid back on the animal, which caused lower received click output levels that were therefore excluded. (d) The swimming effort of the porpoise increased during and after the exposure period. Note that the respiration count after exposure only covers ~3.5 min.

**SV1**. Supplemental video with drone footage from AHD playback exposure of un-tagged porpoises.

**Table S1.** Supplemental table containing data on porpoise behaviour and physiology presented as the 15-minute means before and during exposure: Time in crypsis, click rate, swimming effort and respiration rate. This data was used for calculations of acute exposure effects in Table 1.

|  | **Behavioural and physiological response parameters** *(15-min before exposure and 15 minutes during exposure)* | | | | | | | |
| --- | --- | --- | --- | --- | --- | --- | --- | --- |
| ***Animal ID*** | ***Time in visual crypsis***  *(%)*  ******* | | ***Biosonar***  ***click rate***  *(cpm)* | | ***Swimming effort***  *(m/s^2^)* | | ***Resp. rate***  *(min^-1^)* | |
|  | ***Before exposure*** | ***During exposure*** | ***Before exposure*** | ***During exposure*** | ***Before exposure*** | ***During exposure*** | ***Before exposure*** | ***During exposure*** |
| **HP1** | 80 | 78 | 332 | 203 | 0.23 | 0.27 | 2.5 | 2.2 |
| **HP2** | 59 | 92 | 40 | 317 | 0.30 | 0.38 | 2.7 | 2.9 |
| **HP3** | 64 | 72 | 536 | 12 | 0.37 | 0.49 | 3.2 | 4.2 |
| **HP4** | 60 | 70 | 863 | 629 | 0.39 | 0.27 | 3.9 | 3.3 |
| **HP5** | 63 | 78 | 421 | 198 | 0.41 | 0.73 | 3.0 | 3.1 |
| **HP6** | 73 | 99 | ***¤*** | | 0.28 | 0.67 | 3.2 | 2.8 |

***** Visual crypsis is defined as within 2 m from surface or apparent seafloor as evaluated from depth data and bathymetry.

**¤** The audio-recording on the mid-frequency tag (lower sampling rate) on HP6 was not suitable to detect and quantify porpoise high frequency clicks satisfactorily.
